# Clinical relevance of disrupted topological organization of anatomical connectivity in behavioral variant frontotemporal dementia

**DOI:** 10.1016/j.neurobiolaging.2023.01.004

**Published:** 2023-01-11

**Authors:** Min Chu, Deming Jiang, Li Liu, Binbin Nie, Pedro Rosa-Neto, Kewei Chen, Liyong Wu

**Affiliations:** a Department of Neurology, Xuanwu Hospital, Capital Medical University, Beijing, China; b Beijing Engineering Research Center of Radiographic Techniques and Equipment, Institute of High Energy Physics, Chinese Academy of Sciences, China; c School of Nuclear Science and Technology, University of Chinese Academy of Sciences, Beijing, China; d McGill Centre for Studies in Aging, Alzheimer’s Disease Research Unit, Montreal, Canada; e Banner Alzheimer’s Institute, Phoenix, AZ, USA; f College of Medicine-Phoenix, University of Arizona, Tucson, AZ, USA; g School of Mathematics and Statistics, Arizona State University, Tempe, AZ, USA; h Arizona Alzheimer’s Consortium, Phoenix, AZ, USA

**Keywords:** Behavioral variant frontotemporal dementia, Diffusion tensor imaging, Graph theory, Network, Topology

## Abstract

Graph theory is a novel approach used to examine the balance of brain connectomes. However, the clinical relevance of white matter (WM) connectome changes in the behavioral variant frontotemporal dementia (bvFTD) is not well understood. We aimed to investigate the clinical relevance of WM topological alterations in bvFTD. Thirty patients with probable bvFTD and 30 healthy controls underwent diffusion tensor imaging, structural MRI, and neuropsychological assessment. WM connectivity between 90 brain regions was calculated and the graph approach was applied to capture the individual characteristics of the anatomical network. Voxel-based morphometry and tract-based spatial statistics were used to present the gray matter atrophy and disrupted WM integrity. The topological organization was disrupted in patients with bvFTD both globally and locally. Compared to controls, bvFTD data showed a different pattern of hub region distributions. Notably, the nodal efficiency of the right superior orbital frontal gyrus was associated with apathy and disinhibition. Topological measures may be potential image markers for early diagnosis and disease severity monitoring of bvFTD.

## 1. Introduction

Behavioral variant frontotemporal dementia (bvFTD) was early-onset dementia manifests as predominant neuropsychiatric symptoms and social–executive dysfunction (Neary et al., 1998; Rascovsky et al., 2011). It mainly involves gray matter of prefrontal, anterior temporal, anterior cingulate, anterior insula, striatum, and thalamus (Amanzio et al., 2021; Chu et al., 2021; Clarke et al., 2021; Franceschi et al., 2005; Rabinovici et al., 2007; Rosen et al., 2002; Seeley et al., 2008). Moreover, microstructural white matter degeneration was also observed in the anterior cingulum, fornix, and corpus callosum (Dopper et al., 2014; Lu et al., 2014; Mahoney et al., 2014; Panman et al., 2019). However, abnormalities of disease are not only involved in these discrete brain regions but also characterized by the systematic imbalance of whole-brain connectomes. By modeling networks as graphs through a set of nodes (brain regions) and edges (node-to-node connections), graph theory principles applied to neuroimaging data offer a flexible method to quantitatively describe the topological organization of large-scale brain complex networks (Bassett and Bullmore, 2009; Bullmore and Sporns, 2009; Filippi et al., 2013; He and Evans, 2010).

Metrics of graph theory can reflect characteristic of complex network from different aspects. Hubs were nodes that play significant roles in global information integration, and high-level connectivity between hub nodes together forms a brain center so-called rich club (Bullmore and Bassett, 2011; Bullmore and Sporns, 2009; van den Heuvel and Sporns, 2011). The nodal clustering coefficient measures the cliquishness of connections between nodes and their neighborhoods; the nodal shortest path length quantifies the mean distance or routing efficiency between this node and other nodes in the network; the nodal efficiency characterizes the efficiency of information transfer from a given node to other nodes in the network; The degree for a node reflects its information communication ability in the functional network (Bullmore and Bassett, 2011; Bullmore and Sporns, 2009; Wang et al., 2015). Global metrics measure the global conditions of information transfer among all nodes. The detailed definitions of the graph metrics we used are shown in Supplementary Table 1.

Previous neuroimaging studies on bvFTD have revealed disruption of some topological properties, using modalities such as T1, resting-state functional magnetic resonance imaging, fluorodeoxyglucose positron emission tomography, and single-photon emission computed tomography to construct structural covariance, functional, or metabolic connectivity (Agosta et al., 2013; Filippi et al., 2017; Liu et al., 2022; Malpetti et al., 2019; Ng et al., 2021; Nigro et al., 2021; Reyes et al., 2018; Saba et al., 2019; Sedeño et al., 2016; Sedeño et al., 2017; Vijverberg et al., 2017; Zhou et al., 2021). The brain structural covariance and functional networks of bvFTD patients showed preserved small-worldness organization but alterations in global network properties, such as lower average clustering coefficient, reduced global efficiency, and longer characteristic path length representing impairment in both the integration and segregation of information process (Agosta et al., 2013; Nigro et al., 2021; Saba et al., 2019; Sedeño et al., 2016; Sedeño et al., 2017; Vijverberg et al., 2017). At the local level of the network, disruption of nodal metrics like nodal degree or local efficiency was reported, which was particularly predominant over frontal regions (Agosta et al., 2013; Filippi et al., 2017; Malpetti et al., 2019; Sedeño et al., 2016). In addition, extensive reconfigurations of the nodes were observed in the bvFTD group (Agosta et al., 2013; Liu et al., 2022; Malpetti et al., 2019; Zhou et al., 2021). However, few studies have correlated graph analysis metrics with clinical status, to explore the potential clinical relevance of network graph metrics (Ng et al., 2021; Nigro et al., 2021; Reyes et al., 2018; Vijverberg et al., 2017).

Anatomical networks based on white matter fiber tractography can be delineated using diffusion tensor imaging (DTI) in vivo, which can reflect the information flow among different brain regions more directly than other modalities (Bassett and Bullmore, 2009; Bullmore and Bassett, 2011; Yassa, 2011). This white matter network considers 90 brain regions as nodes and is connected by streamlines as edges. However, research about DTI networks is rare in bvFTD (Cividini et al., 2021; Daianu et al., 2016). One white matter network study targeting rich club networks revealed that bvFTD patients exhibit a greater spread of disruption and more peripheral alterations, particularly in the medial frontal areas when compared with EOAD patients (Daianu et al., 2016). Another study reported extensive disruption of structural connectivity of frontotemporal and parietal networks in bvFTD (Cividini et al., 2021). However, no research of white matter networks comprehensively elucidates whether white matter topological property alterations correlate with clinical measures in bvFTD.

The study aimed to explore clinical relevance of white matter topological network in bvFTD and identify whether it can be a potential image marker for bvFTD For this purpose, we focused on the disruption of the topological architecture of the white matter connectome in bvFTD and its relationship with clinical consequences. Based on the previous research, we hypothesized that the distinct aberrant white matter topological network organization of bvFTD would be associated with the severity of the disease condition.

## 2. Methods

### 2.1. Ethics

The study was conducted following the tenets of the Declaration of Helsinki. The clinical protocols were approved by the ethics committee and local institutional review board of Xuanwu Hospital, Capital Medical University, Beijing, China, and the study was conducted in accordance with relevant guidelines and regulations for the use of human subjects in research. Written informed consent was obtained from all participants or their guardians before the start of the study.

### 2.2. Subjects

Sixty right-handed subjects, including 30 bvFTD patients and 30 healthy control subjects were enrolled from July 1, 2017, to June 31, 2021, from the Department of Neurology of Xuanwu Hospital. All patients were diagnosed as probable bvFTD, according to consensus criteria published in 2011 (Rascovsky et al., 2011). Healthy controls, who were age- and sex-matched to the bvFTD patients, had no cognitive decline complaints, depression, or anxiety and performed within the normal range on neuropsychological tests (Mini-mental State Examination [MMSE] score of ≥24 points and Frontotemporal Lobar Degeneration–Clinical Dementia Rating Scale [FTLD-CDR] score of 0 points).

The exclusion criteria for all participants were (1) any serious neuropsychiatric disorder that could affect cognitive function, such as substance abuse, alcoholism, schizophrenia, tumors, or cerebrovascular disease; (2) standard contraindications for MRI examination; and (3) absence of a reliable informant.

### 2.3. Neuropsychological assessment

Each participant underwent a standardized neuropsychological assessment test battery. Global cognitive screening was performed using the MMSE, and disease severity was assessed using the FTLD-CDR. Executive function was evaluated using the Trail Making Tests A and B (TMT A and B) and Stroop I and II tests. Language skill was assessed using the Boston Naming Test (BNT). The severity of behavioral abnormality was assessed using the Frontal Behavior Inventory (FBI), which is separated into a negative apathy symptom subscale (FBI apathy, first 12 items) and a positive disinhibition symptom subscale (FBI disinhibition, last 12 items).

### 2.4. Image data acquisition and preprocessing

#### 2.4.1. MRI acquisition

An MRI scan was performed with a GE Signa PET/MR 3.0-tesla scanner (GE Healthcare, Milwaukee, WI, USA) at Xuanwu Hospital, Capital Medical University. DTI data were acquired using a spine-choecho planar imaging sequence (repetition time [TR]/echo time [TE] = 16500/97.6 ms) with a b-value of 1000 s/mm^2^, applying diffusion gradients along 30 directions. Seventy axial slices, with no slice gap, were acquired (field of view [FOV] = 220 × 220 mm^2^, matrix = 112 × 112, slice thickness = 2 mm, and number of excitations = 1). The parameters of the T1 data were as follows: TR = 6.9 ms, TE = 2.98 ms, flip angle = 12°, inversion time = 450 ms, matrix size = 256 × 256, FOV = 256 × 256 mm^2^, slice thickness = 1 mm, 192 sagittal slices with no gap, voxel size = 1 × 1 × 1 mm^3^, and acquisition time = 4 min 48 s.

#### 2.4.2. DTI data preprocessing

DTI data were preprocessed using the Pipeline for Analyzing Brain Diffusion Images toolbox (Cui et al., 2013) based on the FM-RIB software library (FSL version 6.0, http://www.fmrib.ox.ac.uk/fsl/). First, intracranial tissue was extracted from b0 images using the Brain Extraction Tool. Then, imaging data were corrected for distortions caused by eddy currents and motion artifacts, and diffusion tensors were further corrected. To determine the microstructural organization of white matter, FA was computed for each voxel in the individual brain coordinate space for each patient. Next, the anatomical networks were constructed. First, T1 was standardized into the Montreal Neurological Institute (MNI) space. Then the FA maps were aligned to their corresponding T1-weighted images using affine transformation. The spatial standardization parameters were used to inversely warp the automated anatomic labeling atlas into the individual space for each subject. Three-dimensional curves (streamlines) characterizing fiber tract connectivity by a deterministic tractography method. Using the 90 regions defined by the warped automated anatomic labeling atlas as the nodes, white matter connectivity was modeled as an unweighted network composed of these 90 nodes. The connection of the anatomical network was defined by the fiber numbers between every node pair. Finally, the anatomical network resulted in a 90 × 90 connectivity matrix for each study participant. A threshold method was used to include real structural connections and avoid a spurious one. To define the network edges, we selected a threshold value for the streamline number. In detail, the network edges were defined as 1 if the streamline number between the 2 regions was larger than the 3, and 0 in all other instances, refer to several previous studies (Gong et al., 2009; Shu et al., 2012; Zalesky et al., 2011).

### 2.5. Graph theory network analysis

Graph theoretical analysis was performed using GRETNA (Wang et al., 2015). First, the anatomical network was sparsified and binarized. We set a sparsity value ranged from 0.05 to 0.5 with each increase by 0.05, to make sure within this range yields connected graphs and all individuals’ network satisfied the criteria for small-world properties (sigma*>*1). Then, the topology of anatomical brain networks was examined using various graph theory-based global and nodal metrics at each sparsity value. The *global network metrics* included the clustering coefficient (C_p_), characteristic path length (L_p_), normalized characteristic path length (Lambda, *λ*), normalized clustering coefficient (Gamma, *γ* ), and small worldness (Sigma, *σ* ); efficiency, including global efficiency (E_g_) and local efficiency (Eloc). The area under the curve of the clustering coefficient (aC_p_), characteristic path length (aL_p_), normalized characteristic path length (aLambda), normalized clustering coefficient (aGamma), and small worldness (aSigma), along with global efficiency (aE_g_), and local efficiency (aEloc) were used for further analysis. *Nodal metrics* included the nodal clustering coefficient (C_p_), nodal shortest path length, nodal efficiency, nodal local efficiency, and degree centrality. We used the area under the curve (AUC) of graph metrics for further analyses. The AUC for each graph parameters (efficiency, clustering coefficient, shortest path length, etc.) can provide a scalar over the entire threshold range, which was calculated by Gretna software.

A network with a small-world property has a normalized path length (*λ* = L_p_^real^/L_p_^rand)^ ≈ 1 and normalized clustering (*γ* = C_p_^real^/C_p_^rand)^ *>* 1, where the superscript “real” indicates the real brain network, while “rand” indicates the corresponding indices calculated for the random network. A brain region was defined as a hub when the degree centrality was ≥1 standard deviation higher than the average of the corresponding measure over the entire network (Agosta et al., 2013). Detailed descriptions of the topological metrics based on the Graph Theoretical Network Analysis Toolbox Reference Manual are shown in Supplementary Table 1 (Wang et al., 2015).

### 2.6. Statistical analysis for group comparison

Statistical analyses were carried out using SPSS 22.0 (IBM Corp., Armonk, NY, USA). Continuous data are represented using mean ± standard deviation values. Dichotomous data are represented as numerical values. For group comparison of demographic data, two-sample Student’s *t*-test (continuous data) and chi-squared test (categorical data) were used, and statistical significance was set at *p <* 0.05. For group comparison of global and local topological properties, an independent two-sample Student’s *t*-test was conducted, using age, sex, and education as covariates and using False Discovery Rate (FDR) for correction. Statistical significance was set at FDR adjusted *p <* 0.05.

### 2.7. Associations between topological parameters and neuropsychiatric scores

Partial correlation analyses were conducted between network metrics and neuropsychological assessment scores, using age, sex, and education as covariates and FDR correction. Statistical significance was set at FDR adjusted *p <* 0.05. Neuropsychological assessment included general status (MMSE), disease severity (FTLD-CDR), behavior symptoms (FBI apathy and FBI disinhibition sub-scales), executive function (TMT and Stroop), and language (BNT).

### 2.8. Structural MRI preprocessing and voxel-based morphometry analysis

Structural images were preprocessed using the computational anatomy toolbox 12 (CAT 12), which is based on statistical parametric mapping 12 (SPM12), and is used in MATLAB (MathWorks, Natick, Massachusetts). Voxel-based morphometry (VBM) preprocessing was performed using the default settings of the CAT12 toolbox and the “East Asian Brains” ICBM template. T1-weighted 3D images were segmented into gray matter (GM), white matter (WM), and cerebrospinal fluid partitions. Subsequently, the GM partitions of each subject in native space were high-dimensionally registered and normalized to the standard Montreal Neurological Institute (MNI) space using diffeomorphic anatomical registration through exponentiated lie algebra normalization. The images were then smoothed using an 8-mm full-width half-maximum Gaussian kernel. The preprocessed structural data were used to perform voxel-wise whole-brain comparisons between the bvFTD and control groups using Student’s two-tailed t-test, with age, sex, and education as covariates. The correction threshold was set at FWE corrected *p <* 0.05.

### 2.9. DTI processing and tract-base spatial statistics

Diffusion tensor imaging data were preprocessed using the Pipeline for Analyzing Brain Diffusion Images toolbox software package mentioned formerly. Briefly, preprocessing involved correction of eddy current and head movement, creating a brain mask, and fitting the diffusion tensor model. Outputs were voxel-wise maps of fractional anisotropy (FA), mean diffusivity (MD), Axial diffusivity (AD), and Radial diffusivity (RD). Tract-based spatial statistics analysis was performed. All participants’ FA, MD, AD and RD data were projected onto a mean FA tract skeleton before applying voxel-wise cross-participant statistics. Voxel-wise statistical analyses were conducted using a nonparametric permutation-based inference tool [“randomize,” part of FMRIB Software Library (FSL)] with the general linear model for statistical modeling. Significance thresholds were set at *p <* 0.05 using the threshold-free cluster enhancement option.

## 3. Results

### 3.1. Demographic data and neuropsychological performance

Detailed demographic data and neuropsychological performance are summarized in Table 1. Thirty bvFTD patients were recruited, including 13 men and 17 women. There were no group differences in age, sex, or years of education between the bvFTD patients and the healthy control group.

Compared to the healthy subjects, bvFTD patients had prominent behavioral problems (all *p <* 0.001), which were signaled by the FBI score of 29.63 ± 7.82 points, an apathy subscale score of 16.33 ± 5.18 points, and a disinhibition subscale score of 13.3 ± 4.38 points. The patients also had a poor neuropsychological performance for general mental status, as shown by a mean MMSE score of *<*24 points and an average FTLD-CDR sum of box score of 9.32 ± 2.95 points. Moreover, executive functions were impaired in the patient group; the TMT-A completion time was 105.81 ± 25.70 s, the TMT-B completion time was 208.63 ± 65.02 s, the Stroop I completion time was 52.75 ± 20.62 s, and the Stroop II completion time was 95.81 ± 41.65 s, respectively.

### 3.2. Global network characteristics

Small-worldness was verified in controls and patients with bvFTD (*γ* was *>*1 and *λ* was approximately equal to 1 for all considered thresholds). Most graph-theoretical metrics were altered in patients with bvFTD compared to controls (Table 2). After FDR correction, the mean values of global efficiency, local efficiency, clustering coefficient, shortest path length, Gamma and Sigma were lower/higher (FDR corrected) in bvFTD patients than controls.

### 3.3. Regional nodal characteristics

#### 3.3.1. Hub regions

**Fig. 1.**
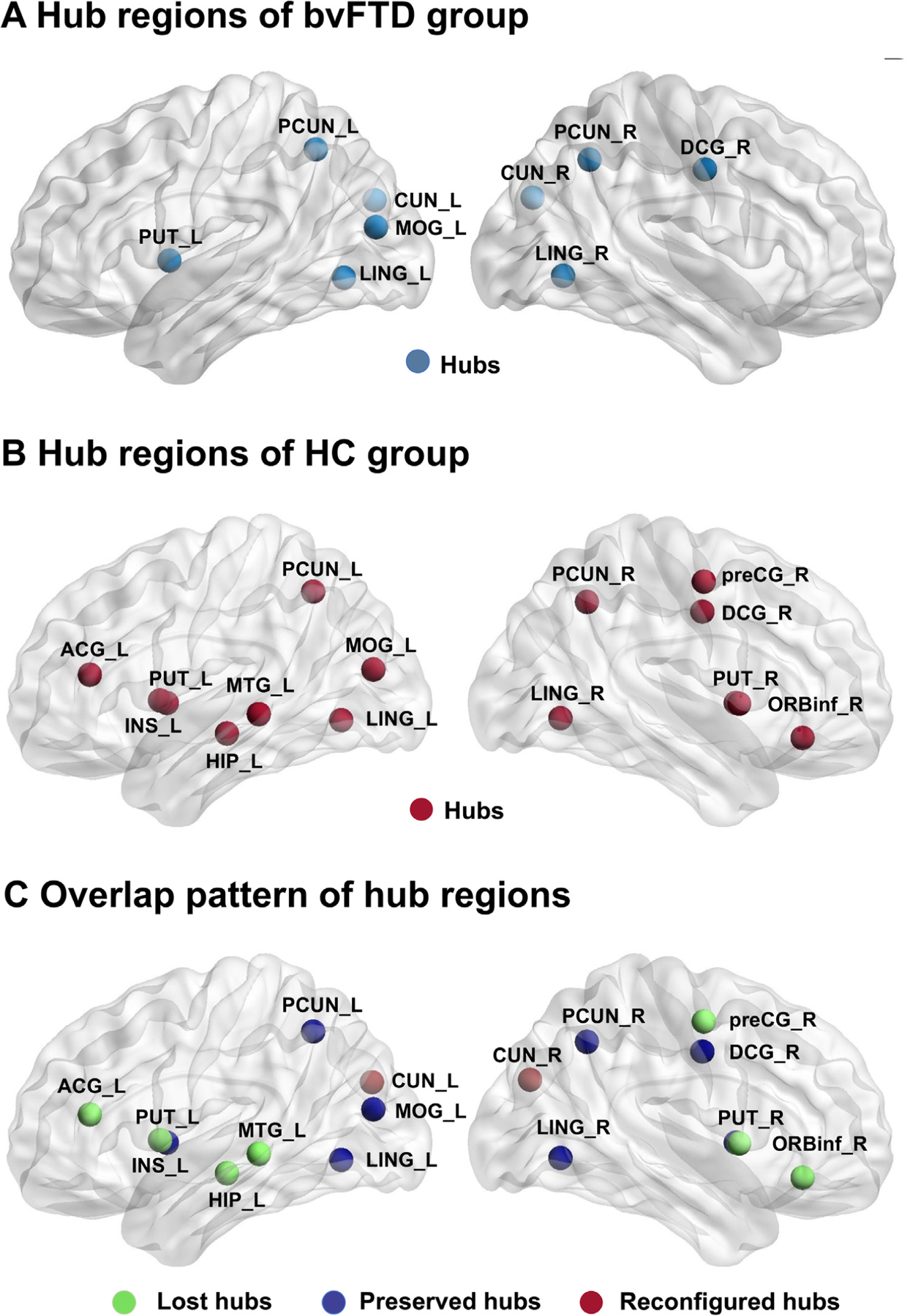
Spatial distribution of hub regions. Brain schematic drawings illustrate the hub regions of behavioral variant frontotemporal dementia (bvFTD) patients (Fig. 1A) and controls (Fig. 1B). Fig. 1C demonstrates the discrepancy of hub regions between the 2 groups. In Fig. 1C, green nodes represent the lost hubs, dark blue nodes represent the preserved hubs, and brown nodes represent the reconfigured hubs. Hubs were identified as brain regions having degree centrality of ≥1 standard deviation greater than the network average. Abbreviations: ACG, anterior cingulate gyrus; CUN, cuneus; DCG, median cingulate and paracingulate gyri; HIP, hippocampus; INS, insula; LING, lingual gyrus; MOG, middle occipital gyrus; MTG, middle temporal gyrus; ORBinf, inferior orbital frontal gyrus; PUT, putamen; PCUN, precuneus; preCG, precentral gyrus; SOG, superior occipital gyrus; L,left; R, right.

Fig. 1A and B shows the hubs in bvFTD patients and controls. Fig. 1C demonstrates the discrepancy of hub regions between the 2 groups. The left anterior cingulate gyrus, left insula, left hippocampus, left middle temporal gyrus, right inferior orbital frontal gyrus, right precentral gyrus, and right putamen were hubs only in the control group and are defined as the lost hubs of bvFTD. The bilateral cuneus were hubs only in the bvFTD group, thus being defined as the reconfigured hub of bvFTD. The left putamen, bilateral precuneus, left middle occipital gyrus, bilateral lingual, and right median cingulate and paracingulate gyri were hubs in both groups and represent the preserved hubs of bvFTD.

#### 3.3.2. Other local network characteristics

Brain regions with abnormal nodal clustering coefficient, nodal local efficiency, and nodal degree centrality are distributed in frontal-temporal-limbic brain regions (FDR corrected). Brain regions with abnormal nodal efficiency are more extensively distributed in frontal, temporal, limbic, parietal, and occipital lobes (FDR corrected). No results were left after FDR correction in analyses of nodal shortest path length. The detailed results were in the supplementary material Table S2–S5, Figure S1–S4.

### 3.4. Correlations between network metrics and neuropsychological test scores

For the node of the *right superior orbital frontal gyrus*, nodal efficiency was negatively correlated with FBI apathy (*r* = −0.6466, FDR adjusted *p* = 0.0006) and disinhibition subscale (*r* = −0.6431, FDR adjusted *p* = 0.0006). No other significant correlations were found.

**Fig. 2.**
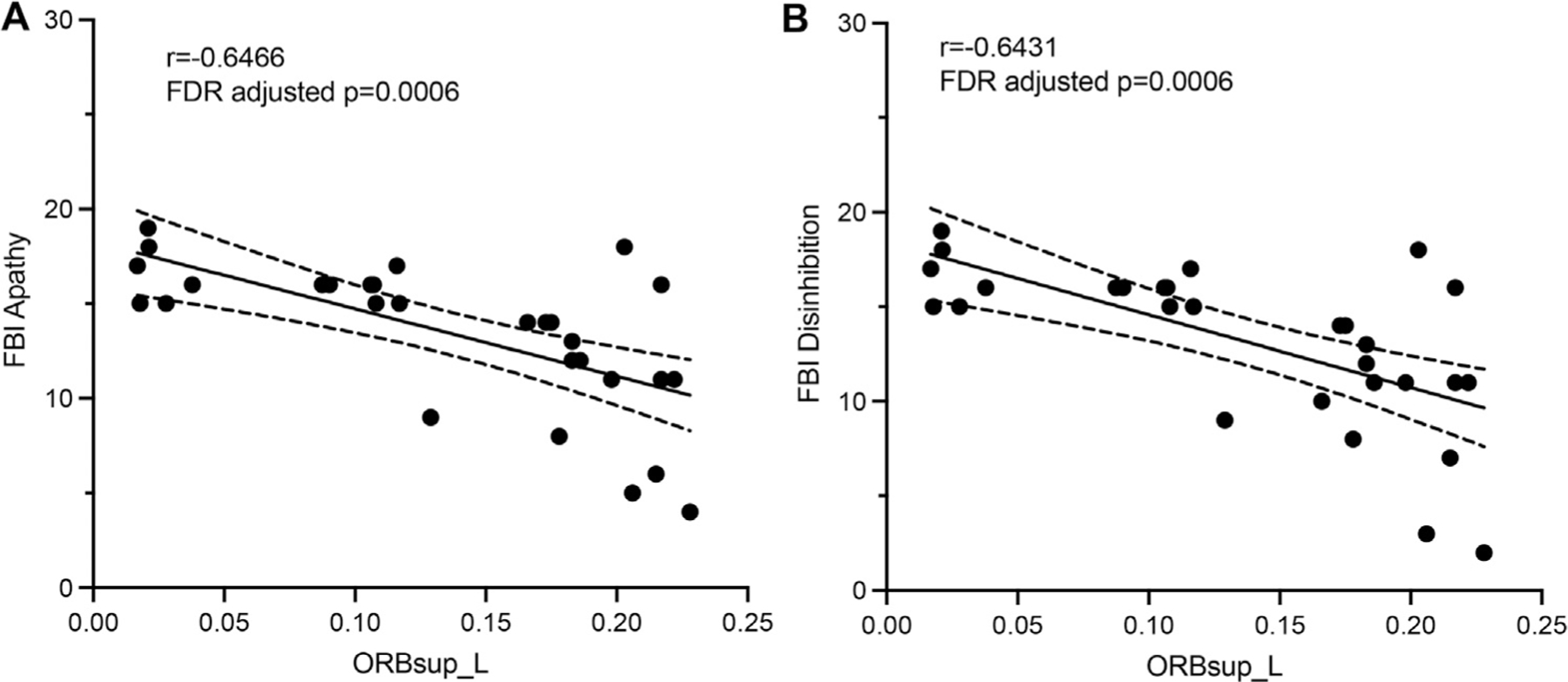
Scatterplots of significant correlations between topological measures of the left superior orbital frontal gyrus (ORBsup.L) and (A) FBI apathy subscale (B) FBI disinhibition subscale.

Scatterplots (Fig. 2) showed significant correlations between topological measures of the left superior orbital frontal gyrus (ORBsup.L) and (Fig. 2A) FBI apathy subscale/ (Fig. 2B) FBI disinhibition subscale.

### 3.5. Gray matter volume and white matter integrity

**Fig. 3.**
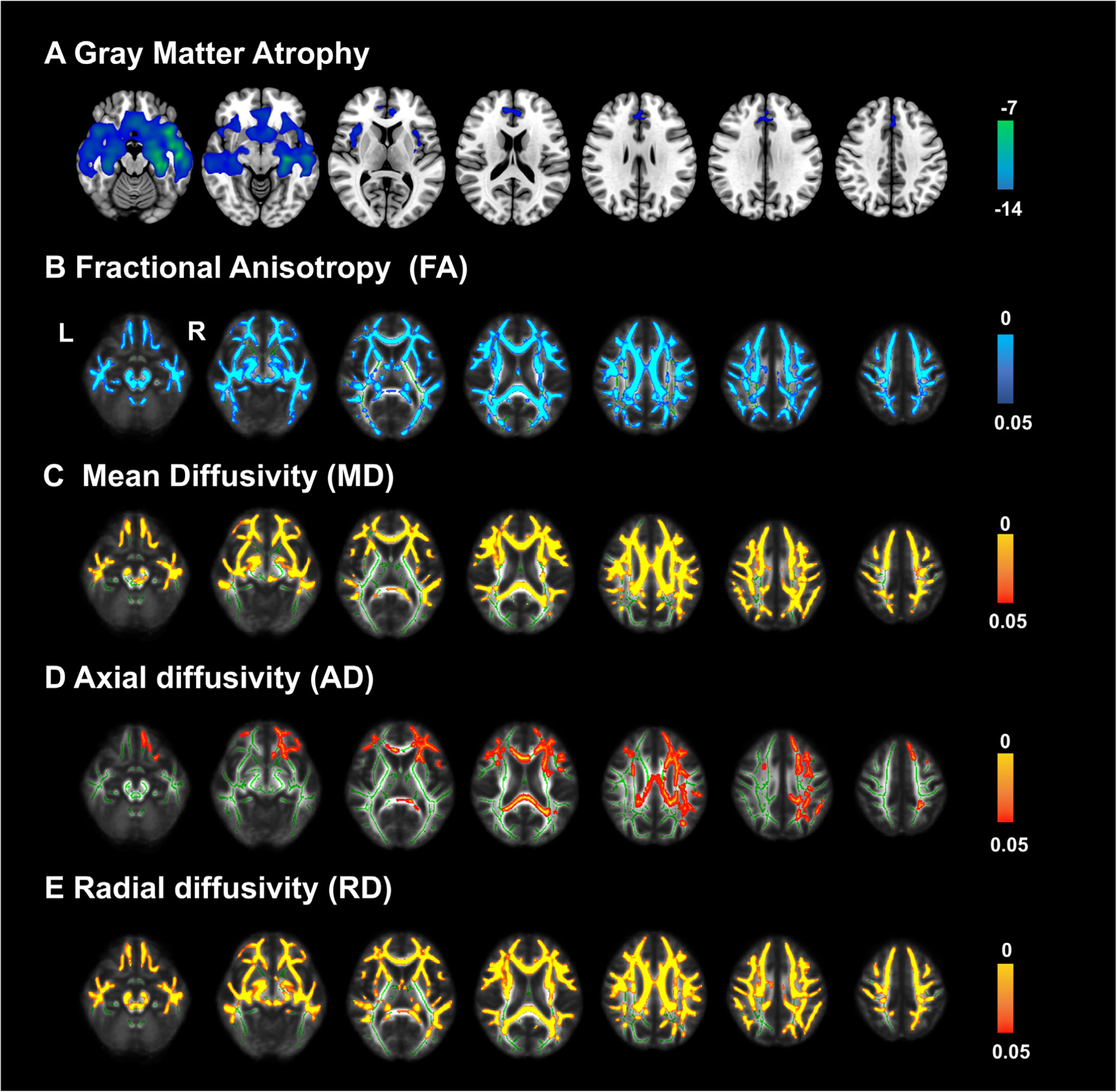
Gray matter atrophy and white matter integrity impairment in bvFTD. Gray matter atrophy pattern (Fig. 1A) is mainly distributed in the orbital frontal, temporal, insula and subcortical regions. Disruption of white matter integrity mainly distributed in frontotemporal white matter fibers, manifesting as decreased fractional anisotropy (FA, Fig. 1B), increased mean diffusivity (MD, Fig. 1B), Axial diffusivity (AD, Fig. 1D) and radial diffusivity (RD, Fig. 1E). Color bar (Fig. 1A) means T value, (Fig.1B–E) means p-value.

Gray matter atrophy in bvFTD group (Fig. 3A) was distributed in the frontal, temporal, and subcortical regions (FWE corrected *p <* 0.05). Detailed information about the brain regions was shown in Supplementary Table S6. The lost hubs were also observed atrophy in VBM analysis. Brain regions in posterior brain regions including parietal and occipital gyrus with preserved volume but disrupted nodal efficiency and degree centrality. Brain regions in anterior brain regions including frontal, temporal and subcortical regions were with gray matter volume loss and disrupted nodal efficiency, nodal local efficiency, nodal clustering coefficient, and nodal degree centrality. The detailed labels of brain regions with disrupted graph metrics but preserved volumes are listed in the Supplementary Table S7.

Fractional anisotropy values (FA, Fig. 3B) were decreased, mean diffusivity (MD, Fig. 1C), axial diffusivity (AD, Fig. 1D) and radial diffusivity (RD, Fig. 1E) values were increased in bvFTD group. The main abnormal regions were distributed in the white matter fibers of frontal, temporal, and parietal, including forceps minor, inferior fronto-occipital fasciculus, anterior thalamic radiation, superior longitudinal fasciculus, uncinate fasciculus, and cingulum (FWE corrected *p <* 0.05). Detailed information about the fiber was shown in Supplementary Table S8.

## 4. Discussion

In this study, graph theoretical analysis was used to capture the underlying disrupted topological organization characteristics of a macroscale anatomical network constructed by DTI tractography in bvFTD patients. Disruptions of the anatomical network in bvFTD patients were identified and correlated with clinical variables. Our findings provide novel insight into the clinical relevance of topological organization of the human brain structural connectome underlying the bvFTD disease spectrum.

Our study targeting DTI topological network provides new insights beyond previous fMRI and T1 data. Different image modalities including structural MRI, functional MRI and DTI can provide diverse information on topological network. DTI networks based on fiber bundles can more directly reflect the anatomical configuration of brain networks ranging from inter-neuronal connectivity to inter-regional connectivity than gray matter volume or cortical thickness. Direct comparisons of structural and functional connectivity in the same cohort of participants suggest that structural connections are highly predictive of functional connections (Bullmore and Sporns, 2009; Honey et al., 2009; Park et al., 2008; Stephan et al., 2000). Structural connectivity of the adult brain is essentially from day to day, but functional connectivity can substantially reconfigure within a few hundred milliseconds (Honey et al., 2009). It can be proposed that anatomical connectivity, as a major constraint of functional connectivity, has a relatively stable and efficient structure to support functional connectivity that is more changeable and flexible (Park et al., 2008).

Most of the brain regions of gray matter atrophy, white matter impairment and graph metric disruption overlapped and distributed in anterior brain regions, which indicates the reliability of graph metrics. We found the lost hubs including the left anterior cingulate gyrus, left insula, left hippocampus, left medial temporal gyrus, right orbital inferior frontal gyrus, right precentral gyrus, and right putamen were also observed atrophy, suggesting its local region dysfunction contribute to the systematic network disruption. Some posterior brain regions such as the parietal and occipital gyrus with preserved volume but disrupted nodal efficiency and degree centrality, which demonstrates the graph metric reflects a systematic function and might be more sensitive than conventional image post-analysis methods.

### 4.1. Global topological metrics and clinical relevance

Small-world properties were found to still exist in bvFTD patients, which indicated that the brain network of patients retained a relatively higher level of integration between distant brain regions and better local communication between neighboring areas compared to the characteristics of random or regular networks, consistent with findings of previous studies on brain structural covariance and functional networks (Agosta et al., 2013; Nigro et al., 2021). However, several of the global graph theoretical metrics were altered, including lower clustering coefficient, higher characteristic path length, reduced global efficiency, and local efficiency in bvFTD patients. This suggests that the wiring cost of brain structural networks in bvFTD patients has been disrupted, and more nodes with fewer connections contribute to the impairment of functional integration.

We found nodal but not global topological metrics significantly correlated with clinical measures. One research of T1 topological network in bvFTD (Nigro et al., 2021) revealed that degree centrality and local efficiency of the caudal anterior cingulate were associated with MMSE scores, but no significant results were found in global metrics, which was similar to our study. Another fMRI topological network analysis in bvFTD (Agosta et al., 2013) only conducted the correlation analysis between global network properties and clinical measures and revealed altered global network properties correlated with executive dysfunction. The discrepancy between studies might be because of the small sample and different image modalities. Further work should be done in a larger group to clarify this discrepancy.

### 4.2. Hub regions and clinical relevance

In the bvFTD group, lost hubs were mainly located in the fronto-temporo-limbic area. Moreover, the preserved hubs and reconfigured hubs were mainly distributed in posterior brain regions, which is in line with the spatial distribution of atrophy, hypometabolism, and pathology of bvFTD (Amanzio et al., 2021; Brettschneider et al., 2014; Chu et al., 2021; ; Clarke et al., 2021; Franceschi et al., 2005; Rosen et al., 2002). We found some discrepancies exist in hub distributions, such as the left inferior temporal gyrus and bilateral lingual gyrus was reported as lost hubs in the fMRI network but not in our DTI network, and some lost hubs such as insula and putamen were not reported in fMRI study(Agosta et al., 2013). These may be due to the varied modality, cohorts, and methods used for data acquisition, preprocessing, or statistical hypothesis testing.

The lost hubs are all critical stations in healthy controls but impaired in bvFTD patients. The insula, especially the anterior insula, has reciprocal connections with limbic regions (Ghaziri et al., 2017), which has been implicated in the integration of emotional, cognitive, and motivational functions and reported significant in bvFTD (Day et al., 2013; Namkung et al., 2017). The anterior cingulate gyrus is a principal hub in the salience network and is in a significant connection region of the Papez circuit, which modulates motivation, goal-directed behaviors, social decision-making, and emotional regulation (Apps et al., 2016; Lockwood and Wittmann, 2018; Rolls et al., 2019). The orbital frontal gyrus is altered in various neuropsychiatric diseases, such as autistic spectrum disorders, and connects with the insula and the anterior cingulate cortex to regulate emotional experiences and executive functions (Schmitz et al., 2006). Subcortical structures such as putamen participate in social behaviors and emotional regulation and may be impaired in the bvFTD disease spectrum (Perry et al., 2017).

The preserved hubs were unimpaired nodes mostly distributed in posterior brain regions. As for the hubs in our healthy control group, there were also some inconsistencies with a previous study; for example, the middle frontal gyrus was previously identified as a hub in human brain functional networks, but we did not detect this hub in our network (Gong, G et al., 2009). However, this may be due to age discrepancies between cohorts as the subjects in our group were generally older. We also found that the bilateral cuneus was reconfigured hubs in the bvFTD group. This may be because the cuneus is not a commonly impaired region of bvFTD patients; it maintains its function and has a more predominant location in the reconfigured networks as a new hub.

### 4.3. Comparison of local properties between groups

The distribution of impaired local properties in nodal clustering coefficient, nodal efficiency, nodal local efficiency, and degree centrality was mainly distributed in the frontal, temporal, and limbic brain regions. Our results are reliable as frontal, temporal, and limbic brain regions overlapped with bvFTD-specific atrophy and hypometabolism pattern and were hubs of the salience network (Chu, M. et al., 2021; Zhou et al., 2010). Two studies reported nodal properties changes in bvFTD using other image modalities. One previous graph theory analysis of functional MRI network showed widespread brain regions with decreased nodal degree centrality, mainly distributed in the medial and dorsal frontal regions, left caudate nucleus and some regions of the insula, temporal, parietal, and occipital gyrus (Agosta et al., 2013); Another structural covariance network study showed altered nodal local efficiency mainly in the middle frontal gyrus, anterior cingulate, precuneus, cuneus, and temporal gyrus, altered nodal clustering coefficient mainly in inferior temporal gyrus (Nigro et al., 2021). The result was not completely consistent because of the small sample, different image modalities and statistical threshold.

In our study, we found orbital frontal gyrus contributes to both apathy and disinhibition. Apathy and disinhibition were the main behavior symptoms in bvFTD of which the neuroanatomical correlates were widely investigated and demonstrated the significant contributions of the prefrontal cortex (Farb et al., 2013; Godefroy et al., 2022; Sheelakumari et al., 2020; Tanguy et al., 2022; Zhou et al., 2021). More specifically, the orbital frontal gyrus was one of the significant brain regions in the frontal-temporal-limbic circuit discovered in previous studies that contribute to apathy using other image modalities (Godefroy et al., 2022; Valotassiou et al., 2022). And it is well-acknowledged that orbital frontal gyrus is linked to impulsive and disinhibition behavior (Migliaccio et al., 2020; Pattij and Vanderschuren, 2020; Tanguy et al., 2022), imbalanced activity in the orbitofrontal cortex and nucleus accumbens was known to disrupt behavior inhibition (Meyer and Bucci, 2016).

Our study has several limitations. First, the sample was relatively small, and a larger homogeneous sample is required to replicate our results. Second, a longitudinal follow-up study is warranted to observe network topological change trajectories and understand how they correspond to symptom progression. Third, our bvFTD phenotypes were defined using stringent clinical diagnostic criteria without pathological verification. Last, we did not explore the brain connectomes from structural covariance, functional and metabolic data, thus, we can’t conclude regarding the potential superiority of white matter anatomical connectivity to explain the clinical status and related variables in bvFTD.

## 5. Conclusion

Our study provides new evidence for the usefulness of using graph theory to capture the characteristics of white matter anatomical connectivity of FTD. Altered nodal graph metrics of the orbital frontal gyrus were correlated with apathy and disinhibition in bvFTD. Mapping the brain structural connectome will help to predict the severity of the behavior symptoms of bvFTD.

## Supplementary Material

Supplementary Material

Editorial certificate

## Figures and Tables

**Table 1:** Demographic data and neuropsychological performance in bvFTD patients (n = 30) and controls (n = 30).

	BvFTD (n = 30)	Controls (n = 30)	*p*-value
Age (years)	62.60 ± 7.16	63.6 ± 5.95	0.572
Sex (male/female)	13/17	13/17	1
Years of education	10.77 ± 4.53	10.50 ± 2.62	0.781
Handness (R/L)	30/0	30/0	
Disease duration (years)	2.20 ± 1.52		
MMSE	16.30 ± 5.17	28.87 ± 1.59	*<* 0.001
FTLD-CDR sum of box	9.32 ± 2.95	0±0	*<*0.001
Executive function			
TMT-A	105.81 ± 25.70	52.97 ± 17.00	*<* 0.001
TMT-B	208.63 ± 65.02	80.57 ± 31.96	*<* 0.001
Stroop I	52.75 ± 20.62	17.47 ± 4.02	*<* 0.001
Stroop II	95.81 ± 41.65	31.40 ± 6.25	*<* 0.001
Language			
Boston naming test	17.10 ± 6.67	26.07 ± 3.18	*<* 0.001
Behavior features			
FBI total score	29.63 ± 7.82	0.20 ± 0.61	*<* 0.001
FBI apathy	16.33 ± 5.18	0.17 ± 0.46	*<* 0.001
FBI disinhibition	13.30 ± 4.38	0.07 ± 0.25	*<* 0.001

Key: bvFTD, behavioral variant frontotemporal dementia; MMSE, Mini-Mental State Examination; FTLD-CDR, Frontotemporal lobar degeneration-Clinical Dementia Rating scale; TMT, Trail Making Test; FBI, Frontal Behavior Inventory; FBI apathy, Frontal Behavior Inventory apathy subscale; FBI disinhibition, Frontal Behavior Inventory disinhibition subscale.

**Table 2:** Comparison of global graph metrics between bvFTD and control group

	bvFTD (n = 30)	Controls (n = 30)	t-value	FDR adjusted *p*-value	Cohen’s d
AEg	0.169 ± 0.052	0.208 ± 0.017	−3.75	0.0009^a^	0.967
AEloc	0.271 ± 0.047	0.308 ± 0.009	−4.21	0.0004^a^	1.089
ACp	0.200 ± 0.029	0.224 ± 0.009	−4.23	0.0004^a^	1.090
ALp	1.438 ± 0.877	1.005 ± 0.076	2.70	0.0106^a^	0.696
aGamma	0.900 ± 0.160	1.016 ± 0.121	−3.12	0.0039^a^	0.804
aLambda	0.477 ± 0.008	0.476 ± 0.014	−0.07	0.5000	0.133
aSigma	0.848 ± 0.143	0.951 ± 0.096	−3.25	0.0035^a^	0.838

Key: aEg, area under the curve of global efficiency; aEloc, area under the curve of local efficiency; aCp, area under the curve of the clustering coefficient; aLp, area under the curve of the characteristic path length; aGamma, area under the curve of the normalized clustering coefficient; aLambda, area under the curve of the normalized characteristic path length; aSigma, area under the curve of small worldness.

a FDR corrected *p <* 0.05.
