# Supplementary material for: Clinical relevance of disrupted topological organization of anatomical connectivity in behavioral variant frontotemporal dementia
Source: Neurobiol Aging. Author manuscript; Available in PMC 2024 May 19. (PMC11102657; doi:10.1016/j.neurobiolaging.2023.01.004)
Supplement: Supplementary Material [file NIHMS1990495-supplement-Supplementary_Material.docx]

**Supplementary Material**

**Supplementary Table S1. Detailed descriptions of the topological metrics according to the Graph Theoretical Network Analysis Toolbox Reference Manual**

| Parameter | Interpretation |
| --- | --- |
| Global metrics | |
| Small world | Small-world networks have a shorter characteristic path length than that of regular networks (high clustering and long path lengths) but greater local interconnectivity than that of random networks (low clustering coefficient and short path lengths). The small-world metric supports both specialized/modularized and integrated/distributed information processing and maximizes the efficiency of information transfer at a relatively low wiring cost. |
| Efficiency | Global efficiency measures the global efficiency of parallel information transfer in a network. The local efficiency of the network measures how efficient communication is among the first neighbours of a given node when it is removed. |
| Nodal Metrics | |
| Clustering coefficient | The clustering coefficient of a given node measures the likelihood its neighborhoods are connected to each other. |
| Shortest path length | The shortest path length of a given node quantifies the mean distance or routing efficiency between this node and all the other nodes in the network. |
| Efficiency | The nodal efficiency for a given node characterizes the efficiency of parallel information transfer of that node in the network. |
| Local efficiency | The local efficiency for a given node measures how efficient the communication is among the first neighbors of this node when it is removed. |
| Degree centrality | The nodal degree for a given node reflects its information communication ability in the functional network. |

**Group comparison of nodal graph metrics**

- **Nodal clustering coefficient**


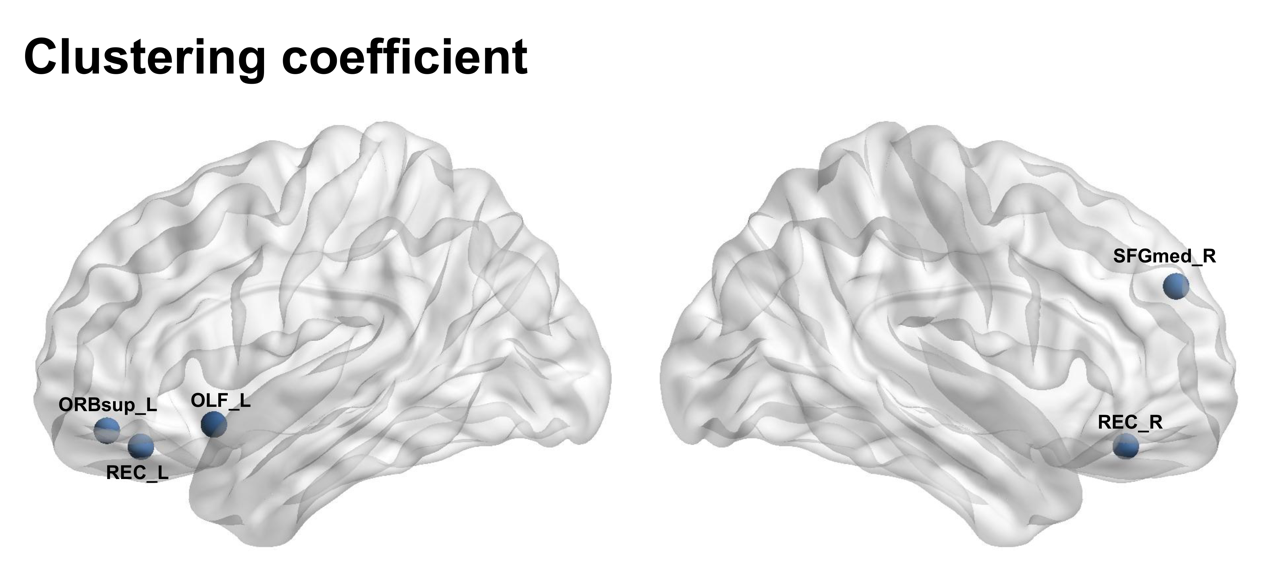


**Figure S1 Nodes with decreased nodal clustering coefficient in bvFTD group.**

Brain regions with altered clustering coefficients in bvFTD compared to controls were shown in Figure S1 and Table S2.

**Table S2 Nodes with decreased nodal clustering coefficient in bvFTD group.**

| Abbreviation | Full name | FDR-adjusted p value |
| --- | --- | --- |
| ORBsup_L | left superior frontal gyrus, orbital part | 0.0016 |
| OLF_L | left olfactory cortex | 0.0457 |
| SFGmed_R | right superior frontal gyrus, medial | 0.0457 |
| REC_L | left rectus gyrus | 0.0208 |
| REC_R | right rectus gyrus | 0.0457 |

- **Nodal shortest path length**

No results left after FDR correction in nodal shortest path length.

- **Nodal efficiency**


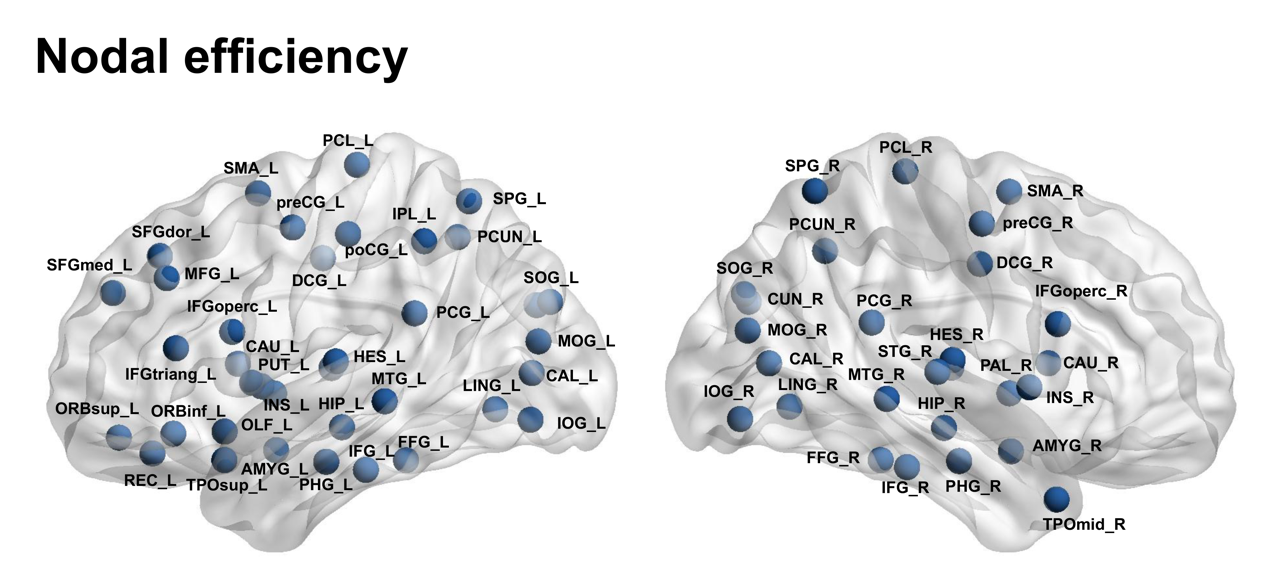


**Figure S2 Nodes with decreased nodal efficiency in bvFTD group.**

Brain regions with altered nodal efficiency in bvFTD compared to controls were shown in Figure S2 and Table S3.

**Table S3 Nodes with decreased nodal efficiency in bvFTD group.**

| Abbreviation | Full name | FDR-adjusted p value |
| --- | --- | --- |
| PreCG_L | left precentral gyrus | 0.0387 |
| PreCG_R | right precentral gyrus | 0.0178 |
| SFGdor_L | left superior frontal gyrus, dorsolateral | 0.0051 |
| ORBsup_L | left superior frontal gyrus, orbital part | 0.0208 |
| MFG_L | left middle frontal gyrus | 0.0062 |
| IFGoperc_L | left inferior frontal gyrus, opercular part | 0.0021 |
| IFGoperc_R | right inferior frontal gyrus, opercular part | 0.0045 |
| IFGtriang_L | left inferior frontal gyrus, triangular part | 0.0081 |
| ORBinf_L | left inferior frontal gyrus, orbital part | 0.0032 |
| SMA_L | left supplementary motor area | 0.0051 |
| SMA_R | right supplementary motor area | 0.0128 |
| OLF_L | left olfactory cortex | 0.0074 |
| SFGmed_L | left superior frontal gyrus, medial | 0.0121 |
| REC_L | left rectus gyrus | 0.0119 |
| INS_L | left insula | 0.0025 |
| INS_R | right insula | 0.0032 |
| DCG_L | left median cingulate and paracingulate gyrus | 0.0087 |
| DCG_R | right median cingulate and paracingulate gyrus | 0.0079 |
| PCG_L | left posterior cingulate gyrus | 0.0051 |
| PCG_R | right posterior cingulate gyrus | 0.0178 |
| HIP_L | left hippocampus | 0.0121 |
| HIP_R | right hippocampus | 0.0150 |
| PHG_L | left para-hippocampal gyrus | 0.0071 |
| PHG_R | right para-hippocampal gyrus | 0.0075 |
| AMYG_L | left amygdala | 0.0060 |
| AMYG_R | right amygdala | 0.0344 |
| CAL_L | left Carine | 0.0071 |
| CAL_R | right Carine | 0.0061 |
| CUN_L | left cuneus | 0.0083 |
| CUN_R | right cuneus | 0.0051 |
| LING_L | left lingual gyrus | 0.0081 |
| LING_R | right lingual gyrus | 0.0121 |
| SOG_L | left superior occipital gyrus | 0.0071 |
| SOG_R | right superior occipital gyrus | 0.0071 |
| MOG_L | left middle occipital gyrus | 0.0086 |
| MOG_R | right middle occipital gyrus | 0.0071 |
| IOG_L | left inferior occipital gyrus | 0.0279 |
| IOG_R | right inferior occipital gyrus | 0.0126 |
| FFG_L | left fusiform gyrus | 0.0071 |
| FFG_R | right fusiform gyrus | 0.0253 |
| PoCG_L | left postcentral gyrus | 0.0279 |
| SPG_L | left superior parietal gyrus | 0.0271 |
| SPG_R | right superior parietal gyrus | 0.0051 |
| IPL_L | left inferior parietal gyrus | 0.0075 |
| PCUN_L | left precuneus | 0.0100 |
| PCUN_R | right precuneus | 0.0051 |
| PCL_L | left paracentral lobule | 0.0074 |
| PCL_R | right paracentral lobule | 0.0129 |
| CAU_L | left caudate nucleus | 0.0051 |
| CAU_R | right caudate nucleus | 0.0087 |
| PUT_L | left putamen | 0.0075 |
| PAL_L | left pallidum | 0.0233 |
| PAL_R | right pallidum | 0.0233 |
| THA_R | right thalamus | 0.0075 |
| HES_L | left Heschl gyrus | 0.0062 |
| HES_R | right Heschl gyrus | 0.0320 |
| STG_R | right superior temporal gyrus | 0.0146 |
| TPOsup_L | left temporal pole, superior temporal gyrus | 0.0157 |
| MTG_L | left middle temporal gyrus | 0.0233 |
| MTG_R | right middle temporal gyrus | 0.0279 |
| TPOmid_R | right temporal pole: middle temporal gyrus | 0.0123 |
| ITG_L | left inferior temporal gyrus | 0.0062 |
| ITG_R | right inferior temporal gyrus | 0.0080 |

- **Nodal local efficiency**


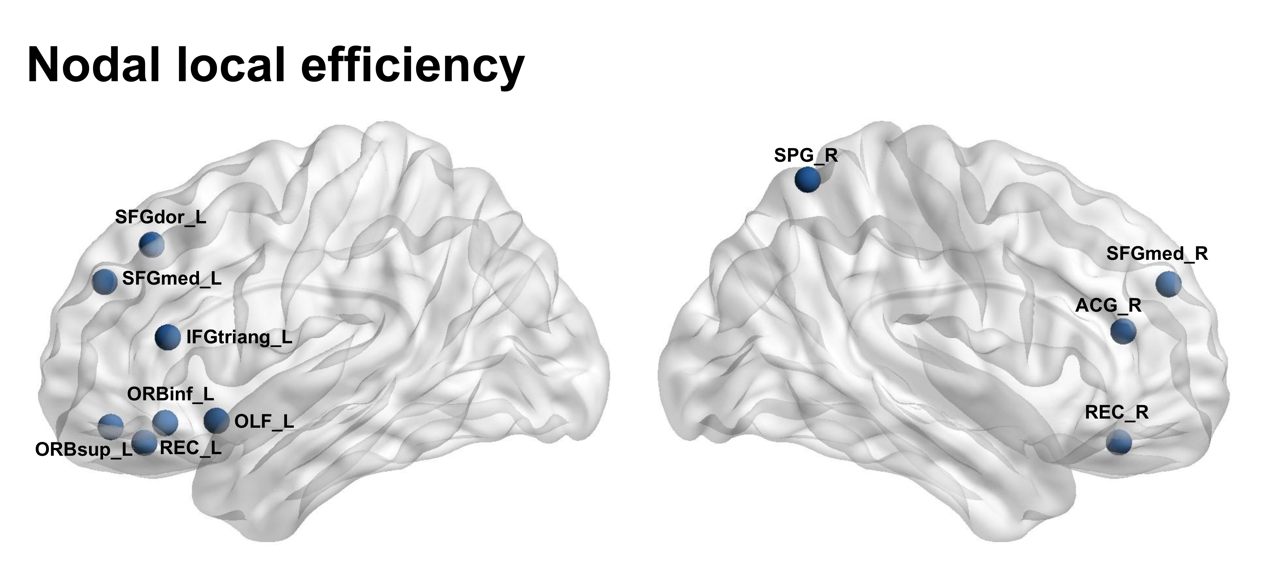


**Figure S3 Nodes with decreased nodal local efficiency in bvFTD group.**

Brain regions with altered nodal local efficiency in bvFTD compared to controls were shown in Figure S3 and Table S4.

**Table S4 Nodes with decreased nodal local efficiency in bvFTD group.**

| Abbreviation | Full name | FDR-adjusted p value |
| --- | --- | --- |
| SFGdor_L | left superior frontal gyrus, dorsolateral | 0.0434 |
| ORBsup_L | left superior frontal gyrus, orbital part | 0.0434 |
| IFGtriang_L | left inferior frontal gyrus, triangular part | 0.0434 |
| ORBinf_L | left inferior frontal gyrus, orbital part | 0.0434 |
| OLF_L | left olfactory cortex | 0.0434 |
| SFGmed_L | left superior frontal gyrus, medial | 0.0434 |
| SFGmed_R | right superior frontal gyrus, medial | 0.0434 |
| REC_L | left rectus gyrus | 0.0434 |
| REC_R | right rectus gyrus | 0.0434 |
| ACG_R | right anterior cingulate | 0.0434 |
| SPG_R | right superior parietal gyrus | 0.0434 |

- **Degree centrality**


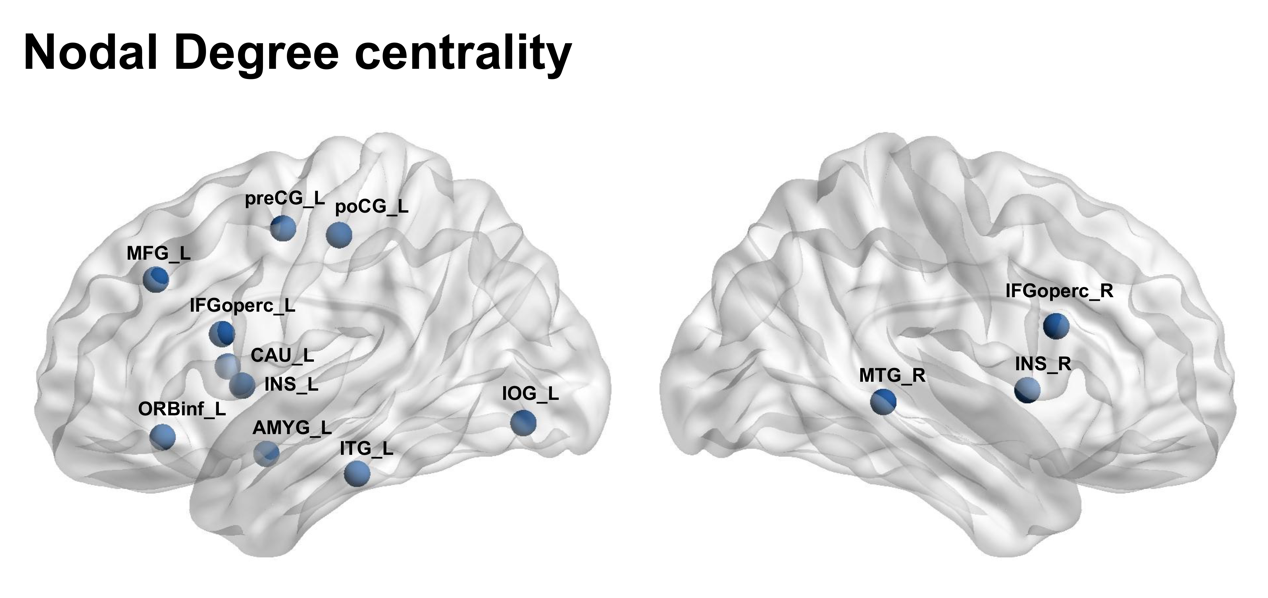


**Figure S4 Nodes with decreased nodal degree centrality in bvFTD group.**

Brain regions with altered nodal degree centrality in bvFTD compared to controls were shown in Figure S4 and Table S5.

**Table S5 Nodes with decreased nodal degree centrality in bvFTD group.**

| Abbreviation | Full name | FDR-adjusted p value |
| --- | --- | --- |
| PreCG.L | left precentral gyrus | 0.0321 |
| MFG.L | left middle frontal gyrus | 0.0336 |
| IFGoperc.L | left inferior frontal gyrus, opercular part | 0.0021 |
| IFGoperc.R | right inferior frontal gyrus, opercular part | 0.0321 |
| ORBinf.L | left inferior frontal gyrus, orbital part | 0.0045 |
| INS.L | left insula | 0.0250 |
| INS.R | right insula | 0.0498 |
| AMYG.L | left amygdala | 0.0336 |
| IOG.L | left inferior occipital gyrus | 0.0336 |
| PoCG.L | left postcentral gyrus | 0.0069 |
| CAU.L | left caudate nucleus | 0.0336 |
| MTG.R | right middle temporal gyrus | 0.0312 |
| ITG.L | left inferior temporal gyrus | 0.0336 |


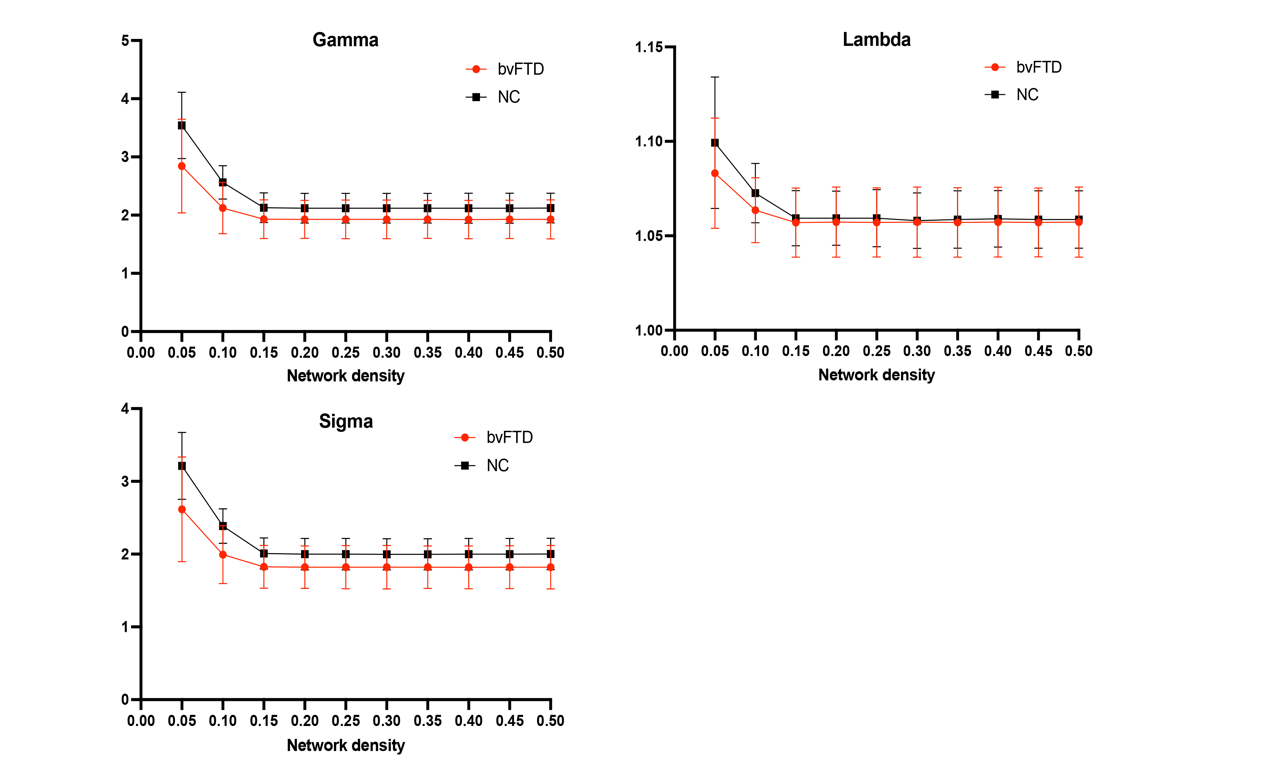


**Figure S5 Small-world parameters (gamma, lambda, and sigma) over the entire threshold range**

**Table S6 Spatial coordinates and peak values of brain areas showing significant gray matter atrophy in bvFTD patients**

| Cluster | Brain region | Peak  intensity | MNI  coordinate | Cluster  size |
| --- | --- | --- | --- | --- |
| 1 | Left: superior temporal pole; middle temporal pole; inferior temporal gyrus; superior temporal gyrus; middle temporal gyrus；Heschl gyrus; insula；hippocampus; para-hippocampal gyrus；inferior frontal gyrus, orbital part；inferior frontal gyrus, triangular part；middle frontal gyrus；olfactory cortex；superior frontal gyrus，dorsolateral part；superior frontal gyrus, medial part；superior frontal gyrus, orbital part; supplementary motor area; median cingulate and paracingulate gyri；rectus gyrus；posterior cingulate gyrus; amygdala; caudate; putamen; pallidum  Right: temporal pole: middle temporal gyrus; middle temporal gyrus; superior temporal gyrus; inferior temporal gyrus; Heschl gyrus; insula; hippocampus; para-hippocampal gyrus; rectus gyrus; superior frontal gyrus, medial; supplementary motor area; anterior cingulate; median cingulate and paracingulate gyri; posterior cingulate gyrus; amygdala; caudate nucleus; putamen; pallidum | -14.47 | -42 6 -36 | 106945 |
| 2 | Right middle frontal gyrus | -5.87 | 24 58.5 3 | 140 |
| 3 | Right thalamus | -6.28 | 1.5 -9 7.5 | 111 |
| 4 | Right inferior frontal gyrus, opercular part | -6.00 | 39 9 28.5 | 210 |
| 5 | Left inferior frontal gyrus, opercular part | -6.06 | -39 12 30 | 195 |

- Hub loss and brain atrophy

The lost hubs including the left anterior cingulate gyrus, left insula, left hippocampus, left medial temporal gyrus, right orbital inferior frontal gyrus, right precentral gyrus, and right putamen were also observed with gray matter atrophy in VBM analysis.

- Nodes with disrupted graph metrics and preserved volumes

The comparison between disrupted graph metrics and gray matter volumes was shown in table S7. We compare the brain regions with disrupted graph metrics with those with gray matter atrophy and divide the disrupted nodes into gray matter volume loss group and gray matter volume preserved group.

We found some posterior brain regions such as the parietal and occipital gyrus with preserved volume but disrupted nodal efficiency and degree centrality. Brain regions in anterior brain regions including frontal, temporal and subcortical regions with volume loss and disrupted nodal efficiency, nodal local efficiency, nodal clustering coefficient, and nodal degree centrality.

**Table S7 Classification of nodes with disrupted graph metrics to volume loss and volume preserved**

|  | Nodes with disrupted graph metrics | Nodes with disrupted graph metrics and loss gray matter volume | Nodes with disrupted graph metrics but preserved gray matter volume |
| --- | --- | --- | --- |
| Nodal efficiency | left precentral gyrus  right precentral gyrus  left superior frontal gyrus, dorsolateral  left superior frontal gyrus, orbital part  left middle frontal gyrus  left inferior frontal gyrus, opercular part  right inferior frontal gyrus, opercular part  left inferior frontal gyrus, triangular part  left inferior frontal gyrus, orbital part  left supplementary motor area  right supplementary motor area  left olfactory cortex  left superior frontal gyrus, medial  left rectus gyrus  left insula  right insula  left median cingulate and paracingulate gyri  right median cingulate and paracingulate gyri  left posterior cingulate gyrus  right posterior cingulate gyrus  left hippocampus  right hippocampus  left para-hippocampal gyrus  right para-hippocampal gyrus  left amygdala  right amygdala  left Carine  right Carine  left cuneus  right cuneus  left lingual gyrus  right lingual gyrus  left superior occipital gyrus  right superior occipital gyrus  left middle occipital gyrus  right middle occipital gyrus  left inferior occipital gyrus  right inferior occipital gyrus  left fusiform gyrus  right fusiform gyrus  left postcentral gyrus  left superior parietal gyrus  right superior parietal gyrus  left inferior parietal gyrus  left precuneus  right precuneus  left paracentral lobule  right paracentral lobule  left caudate nucleus  right caudate nucleus  left putamen  left pallidum  right pallidum  right thalamus  left Heschl gyrus  right Heschl gyrus  right superior temporal gyrus  left temporal pole, superior temporal gyrus  left middle temporal gyrus  right middle temporal gyrus  right temporal pole: middle temporal gyrus  left inferior temporal gyrus  right inferior temporal gyrus | left superior frontal gyrus, dorsolateral  left superior frontal gyrus, orbital part  left middle frontal gyrus  left inferior frontal gyrus, opercular part  right inferior frontal gyrus, opercular part  left inferior frontal gyrus, triangular part  left inferior frontal gyrus, orbital part  left supplementary motor area  right supplementary motor area  left olfactory cortex  left superior frontal gyrus, medial  left rectus gyrus  left insula  right insula  left median cingulate and paracingulate gyri  right median cingulate and paracingulate gyri  left posterior cingulate gyrus  right posterior cingulate gyrus  left hippocampus  right hippocampus  left para-hippocampal gyrus  right para-hippocampal gyrus  left amygdala  right amygdala  left caudate nucleus  right caudate nucleus  left putamen  left pallidum  right pallidum  right thalamus  left Heschl gyrus  right Heschl gyrus  right superior temporal gyrus  left temporal pole, superior temporal gyrus  left middle temporal gyrus  right middle temporal gyrus  right temporal pole: middle temporal gyrus  left inferior temporal gyrus  right inferior temporal gyrus | left precentral gyrus  right precentral gyrus  left Carine  right Carine  left cuneus  right cuneus  left lingual gyrus  right lingual gyrus  left superior occipital gyrus  right superior occipital gyrus  left middle occipital gyrus  right middle occipital gyrus  left inferior occipital gyrus  right inferior occipital gyrus  left fusiform gyrus  right fusiform gyrus  left postcentral gyrus  left superior parietal gyrus  right superior parietal gyrus  left inferior parietal gyrus  left precuneus  right precuneus  left paracentral lobule  right paracentral lobule |
| Nodal  local efficiency | left superior frontal gyrus, dorsolateral  left superior frontal gyrus, orbital part  left inferior frontal gyrus, triangular part  left inferior frontal gyrus, orbital part  left olfactory cortex  left superior frontal gyrus, medial  right superior frontal gyrus, medial  left rectus gyrus  right rectus gyrus  right anterior cingulate  right superior parietal gyrus | left superior frontal gyrus, dorsolateral  left superior frontal gyrus, orbital part  left inferior frontal gyrus, triangular part  left inferior frontal gyrus, orbital part  left olfactory cortex  left superior frontal gyrus, medial  right superior frontal gyrus, medial  left rectus gyrus  right rectus gyrus  right anterior cingulate | right superior parietal gyrus |
| Nodal  Clustering coefficient | left superior frontal gyrus, orbital part  left olfactory cortex  right superior frontal gyrus, medial  left rectus gyrus  right rectus gyrus | left superior frontal gyrus, orbital part  left olfactory cortex  right superior frontal gyrus, medial  left rectus gyrus  right rectus gyrus | None |
| Nodal  degree centrality | left precentral gyrus  left middle frontal gyrus  left inferior frontal gyrus, opercular part  right inferior frontal gyrus, opercular part  left inferior frontal gyrus, orbital part  left insula  right insula  left amygdala  left inferior occipital gyrus  left postcentral gyrus  left caudate nucleus  right middle temporal gyrus  left inferior temporal gyrus | left middle frontal gyrus  left inferior frontal gyrus, opercular part  right inferior frontal gyrus, opercular part  left inferior frontal gyrus, orbital part  left insula  right insula  left amygdala  left caudate nucleus  right middle temporal gyrus  left inferior temporal gyrus | left precentral gyrus  left inferior occipital gyrus  left postcentral gyrus |

**Table S8 Tract-based spatial statistics (TBSS) analysis of bvFTD compared with healthy controls.**

|  | Cluster | Brain region | MNI coordinate | Cluster size |
| --- | --- | --- | --- | --- |
| FA↓ | 1 | Forceps Minor  Left: Inferior Front-occipital Fasciculus, Anterior thalamic radiation, Superior longitudinal fasciculus, Uncinate fasciculus  Right: Inferior frontal-occipital fasciculus, Anterior thalamic radiation, Superior longitudinal fasciculus, Uncinate fasciculus | 7 -49 -57 | 83964 |
| MD↑ | 1 | Forceps Minor  Left: Superior longitudinal fasciculus; Anterior thalamic radiation; Inferior frontal-occipital fasciculus  Right: Superior longitudinal fasciculus, Cingulum. (Cingulate gyrus) | -8 48 -18 | 33527 |
| AD↑ | 1 | Forceps Minor  Left: Superior longitudinal fasciculus, Inferior frontal-occipital fasciculus, Anterior thalamic radiation, Uncinate fasciculus | -19 51 -8 | 14807 |
|  | 2 | Right inferior frontal-occipital fasciculus | 33 46 -6 | 1700 |
|  | 3 | Left Cingulum. (Cingulate. Gyrus) | -17 -49 31 | 210 |
| RD↑ | 1 | Forceps Minor  Left: Superior longitudinal fasciculus, Inferior frontal-occipital fasciculus, Anterior thalamic radiation  Right: Superior longitudinal fasciculus | -46 12 6 | 66664 |
|  | 2 | Left cingulum. (hippocampus) | 5 4 -11 | 257 |

FA fractional anisotropy; MD increased mean diffusivity; AD Axial diffusivity; RD radial diffusivity
