# Supplementary material for: Clinical relevance of disrupted topological organization of anatomical connectivity in behavioral variant frontotemporal dementia
Source: Neurobiol Aging. Author manuscript; Available in PMC 2024 May 19. (PMC11102657; doi:10.1016/j.neurobiolaging.2023.01.004)
Supplement: Editorial certificate [file NIHMS1990495-supplement-Editorial_certificate.pdf]

Charlesworth
Author Services

EDITORIAL CERTIFICATE

This document certifies that the manuscript below was edited for correct English language usage, grammar, punctuation and spelling by qualified native English speaking editors at Charlesworth Author Services.

Paper Title:

Clinical relevance of disrupted topological organization of anatomical connectivity in behavioral variant frontotemporal dementia

Author:

敏 褚

Date certificate issued:

June 16, 2022

cwauthors.com
